# “It’s part of our life now”: a qualitative exploration of the social eating experiences of family members of patients living with head and neck cancer

**DOI:** 10.1007/s00520-022-07427-2

**Published:** 2022-10-28

**Authors:** Mark Dornan, Cherith Semple, Anne Moorhead

**Affiliations:** 1 School of Nursing, Institute of Nursing and Health Research, Ulster University, Belfast, UK; 2 Cancer Services, South Eastern Health and Social Care Trust, Belfast, UK; 3 School of Communication and Media, Institute of Nursing and Health Research, Ulster University, Belfast, UK

**Keywords:** Head and neck cancer, Family, Carer, Social, Eating, Commensality

## Abstract

**Purpose:**

Family members (FMs) of patients with head and neck cancer (HNC) report a change in their social eating experience. They miss out on the opportunities and benefits that eating with others provides. However, few studies investigate FM’s social eating experiences, with existing research primarily focusing on the patient experience. Therefore, the aim of this study was to explore the social eating experiences of FMs of patients who have had treatment for HNC.

**Methods:**

A qualitative research design using semi-structured interviews was used to understand FM’s social eating experiences. Key themes were inductively developed from the data using reflexive thematic analysis.

**Results:**

Twelve interviews were conducted with FMs, and three key themes were identified: (1) changes and challenges experienced by FMs due to HNC patients’ social eating difficulties, (2) living with social eating changes is a balancing act, and (3) FMs’ efforts to promote social eating for a patient with HNC. FMs expressed significant changes to their social eating habits within and outside the home, indicating the need for support to meet their own emotional, psychological and social needs.

**Conclusion:**

FMs experience many demands and tensions, having to balance the psychological impact they experience, which are often minimised, whilst attempting to find the best ways to support, protect and encourage their loved ones to adjust and adapt to social eating changes. Therefore, interventions need to support FMs’ challenges and equip them to know how to best support patients living with HNC and themselves.

## Introduction

Each year, approximately 900,000 patients worldwide are diagnosed with head and neck cancer (HNC), including tumours that affect the upper aerodigestive tract and surrounding structures [1]. Treatment for HNC is often intense and traumatic [2], interposing significantly on vital functions, such as breathing, communication, eating and drinking. Up to 90% of patients experience altered eating and drinking challenges after treatment [3, 4]. Eating and drinking challenges are not limited to physical and functional difficulties but extend to patients’ emotional, psychological and social dimensions of everyday life [5]. For some, this is a diminished or absent social eating experience [6, 7], persisting up to and beyond 5 years after HNC treatment [8]. For some patients, this is due to embarrassment or self-consciousness about how they appear to eat in front of others [9, 10]. Eating with others provides a rich and varied experience to one’s life [11].

Family members (FMs) of patients with HNC report a change in their own social eating habits, eating out of the house less and missing out on their social occasions [12, 13]. This can extend to domestic commensality, where patients and FMs within the same household eat separately and report a loss of togetherness [7, 14]. Existing post-treatment research focuses on the patient, with few studies investigating the FMs’ social eating experiences [7, 10, 15]. Despite a systematic review of social eating experiences for HNC patients [7] identifying FMs as the key support providers, the existing research provides limited insight into how FMs cope or are supported with the multifaceted activities around social eating such as food adaptions, cooking, organising and planning [16]. FMs of HNC patients often do not have access to supportive resources or services and lack appreciation or acknowledgement of the social eating impact [16]. Furthermore, missing out on the opportunities and benefits that eating with others provides placed FMs at risk of reduced quality of life.

Due to limited studies exploring HNC patients’ FMs perspectives on supporting social eating, additional research is required to inform targeted interventions to promote family-centred social eating [15]. This study aims to explore the social eating experiences of FMs of patients who have had treatment for HNC. Objectives are to explore how FMs of patients with HNC:

- Experience changes in their social eating
- Cope with any social eating changes and challenges
- Perceive support received to manage social eating difficulties
- Provide support to patients to promote coping with social eating.

## Methods

### Design

This qualitative descriptive study [17] with FMs was part of a broader research project on the experiences of social eating after HNC treatment with three stakeholder groups: patients [10] relatives and healthcare professionals (HCPs). This study is reported according to the Consolidated Criteria for Reporting Qualitative Research (COREQ) guidelines [18].

### Sampling and sample

A convenient and purposeful sample of FMs of patients living with HNC participated in this study. Inclusion and exclusion criteria were established, displayed in Table 1. FMs were recruited in two ways: (1) patients with HNC who participated in interviews of the corresponding study [10] were asked if they had a FM who would like to participate and (2) alternatively, HNC Clinical Nurse Specialists (CNSs) from three participating healthcare sites in the UK, who were local collaborators for the research, identified potential FMs and sought permission for the researcher (MD) to contact interested participants to extend a research invitation. The study did not require both patients with HNC and FMs to participate. Informed written consent was obtained.

**Table 1:** Inclusion and exclusion criteria of the FMs

Inclusion criteria	Exclusion criteria
• Age > 18 years	• A FM does not have regular contact with the patient
• Next of kin or close relative, including but not limited to a spouse, partner, sibling, parent or child of a person who has completed treatment for HNC	• Unable to speak English
• Able to provide informed consent	• Dementia or cognitive impairment

### Data collection

**Fig. 1:**
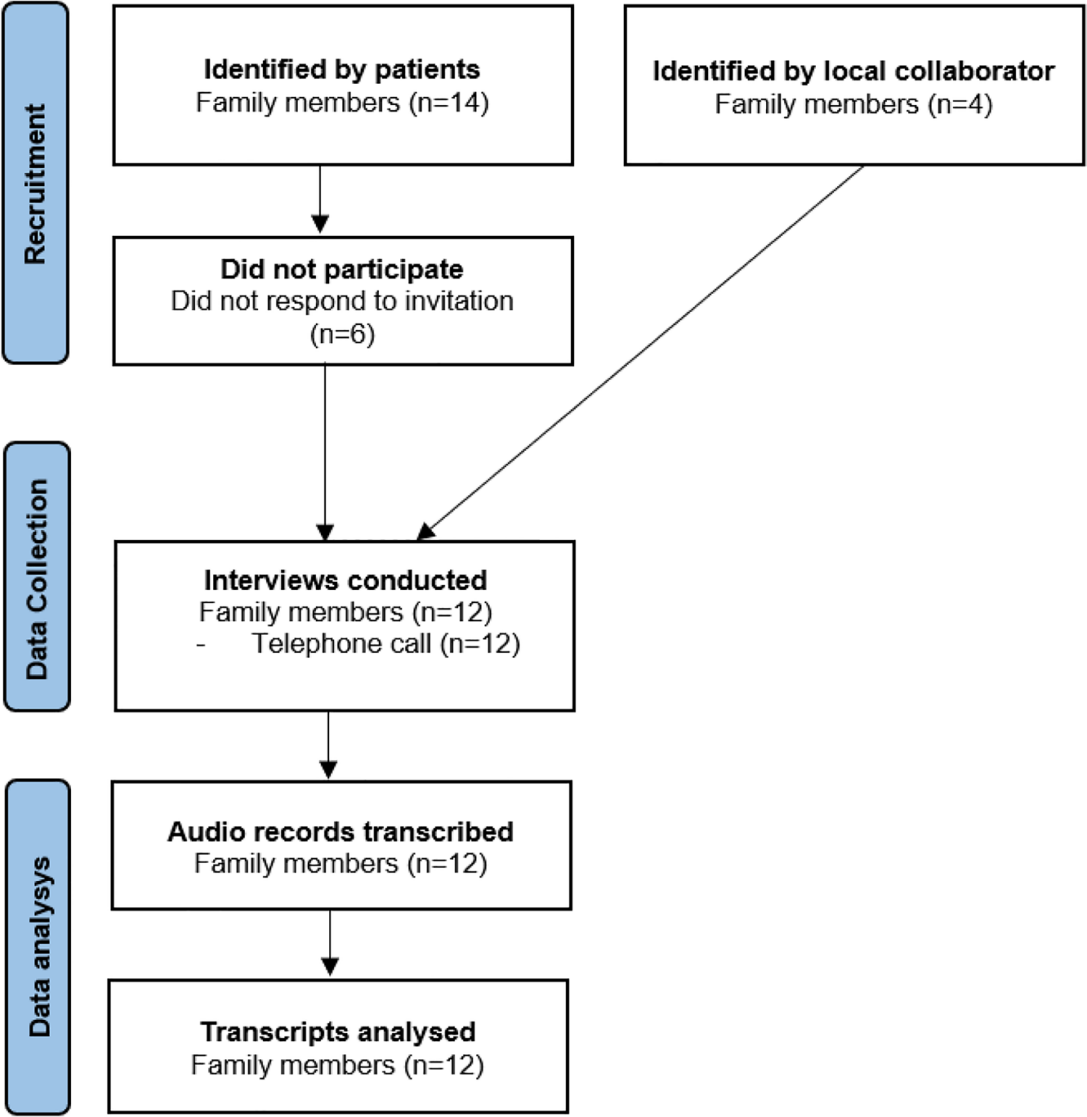
Participant recruitment process

The research team created a topic guide, informed by the literature and subject experts, to support semi-structured interviews with FMs. Individual interviews were conducted from February 2021 to August 2021 via telephone due to COVID-19 restrictions. An online platform was offered but participants opted for telephone interviews. These were undertaken by the first author, a Registered Nurse and academic researcher in HNC survivorship (MD) but not involved in providing care to FMs or their relatives as patients. The first author completed research training in qualitative data collection and analysis. Other team members (CJS & AM) have extensive qualitative research experience. Eighteen FMs were contacted; six did not participate (Fig. 1). FMs who did not participate were non-respondents to the initial invitation or one follow-up email or telephone call.

### Data analysis

Braun and Clark’s six-step approach to reflexive thematic analysis of qualitative data was used [19, 20]. Each recording was transcribed verbatim and reread to ensure familiarity. Next, the transcription was checked for validity by the research team (CJS), and the transcripts were coded by the first author (MD) in NVivo Version 12. As an inductive process, codes were developed and collated by the first author (MD), identifying initial themes. One co-author (CJS) independently read all transcripts and inductively developed codes and themes to ensure rigour and credibility. Through iterative discussion with the research team (MD, CJS, AM), the themes were refined, and final themes were established to ensure the correct meaning of the participants had been captured.

### Ethical considerations

This research study received approval from the NHS Wales Research Ethics Committee (20/WA/0253) and research governance from local participating healthcare trusts within the UK. Due to the nature of the topic and the participant group, a distress protocol was created to ensure appropriate actions or signposting was provided, if applicable, and subsequently required after two FMs raised concerns about a patient. Particular attention was paid to ongoing reflexive practice to promote transparency and trustworthiness. Field notes were taken during the interview, and reflective notes were made after the interview with regular discussions with the research team to assess any potential influences in the data collection and analysis.

## Results

Twelve FMs of patients with HNC participated in the study, nine women and three men representing various relationships, including wife, husband, partner, mother, daughter, sister and niece (Table 2).

**Table 2:** Characteristics of participating FMs

FM ID	Gender (*F* = female, *M* = male)	Age (years)	Relationship to the patient with HNC	Lives with patient	Patient’s initial tumour	Patient’s treatment	Time since patient’s treatment (years)
1	F	61–75	Wife	Yes	Oral cavity	Surgery	2–5
2	F	46–60	Sister	No	Oral cavity	Surgery & radiotherapy	2–5
3	F	46–60	Wife	Yes	Oral cavity	Surgery & radiotherapy	2–5
4	F	46–60	Niece	No	Oral cavity	Surgery & radiotherapy	2–5
5	F	46–60	Daughter	No	Oral cavity	Surgery & radiotherapy	2–5
6	M	61–74	Partner	Yes	Oral cavity	Surgery & radiotherapy	2–5
7	M	18–30	Husband	Yes	Oral cavity	Surgery & radiotherapy	5 +
8	F	46–60	Mother	No	Oral cavity	Surgery & radiotherapy	5 +
9	F	31–45	Wife	Yes	Oral cavity	Surgery, radiotherapy & chemotherapy	1–2
10	F	46–60	Wife	Yes	Throat	Surgery, radiotherapy & chemotherapy	5 +
11	M	46–60	Husband	Yes	Throat	Surgery, radiotherapy & chemotherapy	2–5
12	F	46–60	Wife	Yes	Oral cavity	Surgery, radiotherapy & chemotherapy	2–5

Three themes were identified: (1) changes and challenges experienced by FMs due to HNC patients’ social eating difficulties, (2) living with social eating changes is a balancing act and (3) FMs’ efforts to promote social eating for a patient with HNC.

### Theme 1: Changes and challenges experienced by FMs as a result of HNC patients’ social eating difficulties

Changes to social eating for FMs and associated challenges made daily life look different both 1.1 within the family home and 1.2 outside of the home.

### Subtheme 1.1: Within the family home

FMs often reported changes surrounding mealtime routines at home, including eating alone as the patient did not want to eat with others. Commonly FMs facilitated and adjusted mealtimes by eating at a different time, cooking more than one meal or incorporating food modifications. For FMs, dealing with changed social eating patterns at home was often challenging and frustrating.

we would have always sat at the table and had our breakfast and our dinners together, then lunches together at the weekends. That all, that all changed, we find different ways of doing things and we eat at different times of the day, to facilitate that. (FM11).

Frequently mealtimes focused on the physical activity of eating or the nutritional value of food, which was disruptive to the social experience and flow of communication. Eating required concentration for patients, so conversation was a secondary consideration at mealtimes. FMs reported how patients would have to wait to finish their swallow before attempting to speak.

It might take say, could take her five or six seconds to swallow a mouth full of food […] but then after that she's quite capable of having a conversation but yeah, there would be a lot of concentration from her point of view. (FM11)

Social eating challenges at home were not limited to those living within the home. For example, it was disappointing that certain relatives and friends could not be invited to eat for celebrations due to a patient’s embarrassment of eating with others.

well we stopped having Christmas, we just stopped them sort of things. (FM1)

At home, it was noted that if patients came to the dinner table, FMs frequently finished their meal earlier, but some continued sitting at the table to participate in the conversation, and others left the table to permit their loved ones’ time to finish the meal at their own pace. Alternatively, some FMs slowed their eating to continue the eating activity together.

### Subtheme 1.2: Outside the home

Although the home eating dynamic often changed following a patient’s HNC treatment, eating together outside the home was also profoundly impacted. Challenges were identified when going to restaurants, hotels and also the homes of other FMs or friends.

definitely been challenging, it's been quite stressful […] simply going to a restaurant has been, it's just so difficult, you know any other couples just walk on and, and just sit down and have a meal together and walk out… that's totally different. (FM7)

For some patients and FMs, all eating was completed at home, both in the short term and longer term, leading to significant changes in relatives’ social lifestyles; considered as both a challenge and a loss.

I think we ate out once on the way home from treatment at the start of it, an appointment but there was nobody else in the place. And then we tend to be now, like as a family for the day I tend to pack a picnic, you know, have coffee in a flask. Something like that there so we're not going in anywhere. (FM9)

Eating outside of the home for FMs induced feelings of stress surrounding the uncertainty of available suitable food for patients and the potential lack of awareness and understanding from hospitality staff.

### Theme 2: Living with social eating changes and challenges is a balancing act

Living with social eating changes and challenges was a balancing act for FMs, indicated by the following two subthemes: 2.1 balancing emotions and 2.2 minimal attention to the needs of FMs.

### Subtheme 2.1: Balancing emotions

At times, relatives felt overwhelmingly emotional whilst witnessing some of the physical challenges their loved ones endured when eating together or alone, reporting feelings of upset and powerlessness to help them. Furthermore, some FMs felt guilty about cooking or eating their meals before patients.

I feel guilty for eating a meal in front of him because he can't eat and then the smell of the food as well in the house I just felt so guilty all the time cooking. (FM12)

FMs described how they felt sad and disappointed that their own social life had changed, consequently missing out on social eating occasions with friends and family. Reasons provided included not bring able to attend with their loved ones or not wanting to go independently and leave them at home. This was poignant if significance was attached to the social eating occasion such as a birthday or anniversary.

You know, that's, that's sad to think, you know that, you know, where families just maybe on a Saturday night or if a birthday comes up or something they just book a meal and on they go. (FM3)

Sometimes there was personal tension for FMs, mainly if they perceived they were not fulfilling their role of being a supportive caregiver. Often FMs expressed concern about providing inadequate support for HNC patients, typified and exemplified as they felt ill-equipped for the role.

### Subtheme 2.2: Minimal attention given to the needs of FMs

To balance and minimise the pressure on the patient around social eating, FMs would diminish their own needs, wants and desires by eating something they enjoyed less or altering their own eating habits, for example not visiting certain restaurants or eating certain foods on holiday.

Yeah, a lot of restaurants I've never been to because it's just too messy for my [partner] to even think about, you know, like Wagamama where you can go and have noodles and stuff. [Partner] would never even think about going there. (FM7)

FMs perceived that healthcare interactions focused on the patient and seldom incorporated their opinions within the consultations. FMs noted that they were not directly offered support by clinical teams but signposted to charities to access support for themselves.

and they offered [Macmillan] and I said no thank you. I appreciate it. That's very kind (FM5)

If there were children within the home, FMs also had to balance the potential impact of changed family mealtimes to minimise the loss of time spent together as a family. The FM may have to support social eating for the patient and children, thus limiting the attention on their own needs and requirements.

### Theme 3: Family members’ efforts to promote social eating for patients with HNC

#### Theme 3.1: Protecting

To support HNC patients’ social eating challenges, FMs described how they had developed skills, tools and strategies with limited input from others, particularly the HCPs involved in acute care. For example, emotional support, finding appropriate restaurants and focused consideration on food safety.

And whenever he was ready to go out uhm it would have been just before we went out for something to eat, you know, for a trial and error trying to find out what he can eat or where we can go to eat, you know those kind of things. (FM12)

FMs expressed having to balance multiple approaches to assist with the multifactorial needs associated with social eating difficulties, which often changed over time. Sometimes, relatives, despite their best efforts to protect the patient by providing physical and emotional support, repeatedly got it wrong and subsequently were dealing with emotional sequelae of this.

I made her level four dinner that day and I made sure the chicken was level four, but I got it wrong […] so I got it wrong and she cried, I cried, my daughter all, I felt like I'm really disappointed her, and let her down you know (FM18)

Protection encompassed FMs often becoming an advocate for the patient in restaurants where they would speak to hospitality staff in advance to ensure adequate provision in place or for patients with altered speech, such as post-laryngectomy or if patients were too embarrassed to speak up for themselves.

#### Theme 3.2: Promoting and encouraging social eating

FMs reported a ‘balancing act’ between encouraging patients to participate in activities and social eating events but not wanting to pressure them to do something they did not want or felt unable to do. For example, some FMs let the patient control the social eating situation, following their cues, requests or merely offering suggestions. It was important for FMs to find the balance between knowing the patients’ needs and abilities to help them provide the appropriate support.

Sometimes finding the right approach or finding out what works best took time, developed through trial and error, significantly as patients’ needs and preferences could change over time.

I think you just have to kind of just have to go with the flow. You have to just go with it, I think, things will change you know, regimes change, attitudes change (FM11)

Some FMs took an alternative approach, directly representing what they felt was best for the patient. This action was reported as an expression of devotion and care for the patient in taking control of the situation. Others insightfully recognised that this approach led to negative consequences such as reducing patients’ self-esteem. An improved outcome was when FMs remained balanced in their approach to fluctuation between encouragement, guidance and providing choices. FMs also recognised patients’ need to overcome psychological challenges when eating in front of others and had to encourage not solely the physical act of eating food but also the mental attitude towards eating with others.

## Discussion

This study uniquely highlights the specific social eating challenges, within and outside of the home, for FMs of patients living with HNC including limitations on independent social eating opportunities, reduced enjoyment in social eating activities and additional carer burdens. Our findings clearly demonstrate that FMs have to balance the significant social eating support required by their loved ones, whilst minimising and curtailing their own emotional and social needs, often with little support and input from HCPs.

Everyday sociability and activities considerably change for partners of patients with HNC [21, 22]. This study’s specific focus on social activities for FMs that occurred around food or eating and drinking, identified that FMs socialise less due to the patient’s functional and psychological changed relationship with food. Preparing suitable food at home and finding accommodating restaurants alongside the patient’s reduced confidence and motivation to eat with others were barriers to FMs social eating experience. A key addition from this research indicates how post-treatment challenges for patients can impact a FM’s social eating experience inside and outside the house. Whilst much socialising in today’s contemporary society revolves around food and drink [23], the significant salient expression remains in the everyday, commonplace occurrence of eating at home [24].

Family meals are the most critical social eating occasion [25]. This was identified by FMs in this research, notably recognised by those who had young children within the home. Due to the massive impact altered social eating had on everyday life, those with children in the home attempted to minimise the impact on them by endeavouring, where possible, to continue to eat together as a family. This may have been without the patient or sometimes having to organise family social occasions without food. Cognisance given to family meals is essential for the development of formative behaviours with family bonds created around shared meals and challenges can negatively impact family experiences, memories and holidays [11, 26, 27]. Whilst there are recognised emotional and social changes in children who have a parent living with cancer [28], further research should consider the impact of social eating challenges on children living with a parent who has altered eating after HNC treatment.

In addition to FMs socialising less due to a patient’s altered social eating, FMs socialised less due to focusing on the patient’s needs. As a result, FMs often underreport their own needs, focusing on the patient’s needs [29]. Focusing on the patient’s needs was apparent within this study as FMs often overlooked their own experiences to give an account of their loved ones’ challenges. Recent research [30] with FMs of patients with HNC summarises the sentiments as their “life is not their own”. An increase in post-HNC treatment caregiving activities contributed to less attention on oneself such as eating simpler, less appetising meals at alternative times [31] and having to balance a multitude of tiring and stressful tasks such as the coordination of healthcare appointments, activities of daily living and cooking to facilitate altered eating functions, planning and trialling a variety of adjustments [32–34]. It is noteworthy to consider a trial-and-error approach to eating in restaurants may not be a financially viable option. Purchasing meals that subsequently cannot be eaten as families can add to the already recognised financial burden of patients with cancer [35].

FMs reported a plethora of emotions; guilt hence eating alone or having to eat secretly to ensure their nutritional and support needs were met [26]; disappointment when unable to invite others to eat at their home or frustration when uncertain about how best to provide the correct psychological and practical support. Not all FMs in this sample ate alone; however, some FMs identified that they sometimes had to manage the loss of potential events or holidays they forsook or restaurants and foods they forwent. Previous commensality research describes a diminished quality of life and the sense of loss of no longer having someone at home with whom to share a meal [31, 36]. The negative impact of ongoing eating alone can be lonely, which places people at risk of poorer mental health, anxiety, stress, social isolation and overall subordinate health outcomes for FMs and patients [37]. Finding new commensal networks may promote independent quality of social life [36]. However, in this research, FMs suggested they were unlikely to go out to eat without their loved ones or go to a wedding alone. FMs may limit their social life to support the patient’s emotional needs and may need encouragement to maintain a social life while in a caring role [37].

Eating and nutrition has been described as torturous after cancer treatment and individually distressing for patients and their family [38]. Yet FMs reported that they did not speak to healthcare professionals about the challenges of social eating and were often forgotten about in the healthcare consultation. FMs depict significant unmet psychological, information and physical needs and a lack of involvement in healthcare planning [39]. In addition, to providing vital and pivotal patient support, FMs must also be afforded ongoing support and future supportive interventions. This is fundamentally important, as FMs are the key support providers for HNC patients promoting positive post-treatment social eating coping [7, 10] plus the evident need to equip FMs with coping strategies for their own needs. FMs of patients with additional requirements, such as those with laryngectomies or feeding tubes, may be at particular risk of socialising less than before treatment and require further research and targeted supportive measures [40].

### Limitations

Several FMs did not respond to an initial invitation (*n* = 6); therefore, participants who took part may have had a particular interest in sharing their experiences about social eating. Data collection took place remotely during the COVID-19 pandemic; although it was not thought that this altered the richness of the data collected, beneficially minimised further burden on FMs to attend another appointment. The data collection period was during the COVID-19 pandemic when people ate less socially with others; therefore, participants had to remember what the experience of social eating was like or anticipate what their future experiences may be. This could be considered a small study (*n* = 12) of FMs; a larger sample size may glean a broader range of experience, but recruitment ceased due to the richness of the data capture addressing this study’s aim.

### Clinical implications

HCPs must be aware that social eating challenges affect patients and FMs after HNC treatment. It is imperative that FMs’ unique needs and challenges are recognised, and appropriate support provided. Supporting FMs’ changes with social eating may not be limited to one profession, and an interdisciplinary approach should be taken to recognise and support social eating challenges.

## Conclusion

Social eating, at home and outside of the home, is impacted for FMs of HNC patients due to multifactorial changes and challenges encountered after treatment. As a result, FMs experience many demands and tensions, balancing the psychological impact they experience, which are often minimised, whilst attempting to find the best ways to support, protect and encourage their loved ones to adjust and adapt to social eating changes. Therefore, interventions need to support FMs’ challenges and equip them to know how to best support patients living with HNC.

## Data Availability

N/A.

## Abbreviations

HNC: Head and neck cancer
FM: Family member
HCP: Healthcare Professional
